# Evaluation of transgene expression characteristics and DNA vaccination against melanoma metastasis of an intravenously injected ternary complex with biodegradable dendrigraft poly-L-lysine in mice

**DOI:** 10.1080/10717544.2021.1895904

**Published:** 2021-03-09

**Authors:** Yukinobu Kodama, Ayako Tokunaga, Junya Hashizume, Hiroo Nakagawa, Hitomi Harasawa, Tomoaki Kurosaki, Tadahiro Nakamura, Koyo Nishida, Mikiro Nakashima, Mitsuru Hashida, Shigeru Kawakami, Hitoshi Sasaki

**Affiliations:** a Department of Hospital Pharmacy, Nagasaki University Hospital, Nagasaki, Japan; b Graduate School of Biomedical Sciences, Nagasaki University, Nagasaki, Japan; c Graduate School of Pharmaceutical Sciences, Kyoto University, Kyoto, Japan

**Keywords:** Splenic vector, DNA vaccine, ternary complex, melanoma, gene delivery

## Abstract

We developed a biocompatible splenic vector for a DNA vaccine against melanoma. The splenic vector is a ternary complex composed of plasmid DNA (pDNA), biodegradable dendrigraft poly-L-lysine (DGL), and γ-polyglutamic acid (γ-PGA), the selective uptake of which by the spleen has already been demonstrated. The ternary complex containing pDNA encoding luciferase (pCMV-Luc) exhibited stronger luciferase activity for RAW264.7 mouse macrophage-like cells than naked pCMV-Luc. Although the ternary complex exhibited strong luciferase activity in the spleen after its tail vein injection, luciferase activity in the liver and spleen was significantly decreased by a pretreatment with clodronate liposomes, which depleted macrophages in the liver and spleen. These results indicate that the ternary complex is mainly transfected in macrophages and is a suitable formulation for DNA vaccination. We applied the ternary complex to a pUb-M melanoma DNA vaccine. The ternary complex containing pUb-M suppressed the growth of melanoma and lung metastasis by B16-F10 mouse melanoma cells. We also examined the acute and liver toxicities of the pUb-M ternary complex at an excess pDNA dose in mice. All mice survived the injection of the excess amount of the ternary complex. Liver toxicity was negligible in mice injected with the excess amount of the ternary complex. In conclusion, we herein confirmed that the ternary complex was mainly transfected into macrophages in the spleen after its tail vein injection. We also showed the prevention of melanoma metastasis by the DNA vaccine and the safety of the ternary complex.

## 1. Introduction

Vaccines have potential in the prevention and treatment of several cancers. However, traditional protein-based vaccines have been limited by the weak induction of cell-mediated immune responses (Plotkin, 2005). Furthermore, inactivated and subunit vaccines are often impotent, stimulating suboptimal immune responses that are incapable of achieving therapeutic effects against cancer (Karch & Burkhard, 2016). Conventional vaccine technologies have detrimental disadvantages. On the other hand, DNA vaccines have many advantages over traditional vaccines, such as safety, versatility, easy production, and cost-effectiveness (Klinman et al., 2010). Then, DNA vaccines have been attracting increasing attention. DNA vaccines induce both humoral and cellular immunity. However, naked DNA vaccines degrade in the body and, thus, transfection efficiency in APCs, such as macrophages and dendritic cells, is markedly reduced (Gautam et al., 2000). To overcome this issue, various vectors for DNA vaccine delivery are now being developed. Cationic polymers and liposomes as non-viral vectors are capable of delivering nucleic acids but are cytotoxic due to their slow degradability and accumulation within cells or tissues (Hyoudou et al., 2004).

We previously developed anionic ternary complexes consisting of plasmid DNA (pDNA), cationic compounds, and anionic compounds (Kurosaki et al., 2009; Kodama et al., 2015; Iwanaga et al., 2017; Kodama, Hanamura, et al., 2018; Kodama, Nishigaki, et al., 2018). Anionic ternary complexes consisting of pDNA, polyethyleneimine (PEI), and γ-polyglutamic acid (γ-PGA) exhibited strong gene expression in the splenic marginal zone, which is abundant in antigen-presenting cells (APCs), following their intravenous administration (Kurosaki et al., 2013). Therefore, we applied ternary complexes to the pUb-M melanoma DNA vaccine. The intravenous injection of a ternary complex including pUb-M suppressed the proliferation and metastasis of B16-F10 mouse melanoma cells (Kurosaki et al., 2013). However, this ternary complex is not yet clinically applicable because PEI is not biodegradable in the body, exhibits strong cytotoxicity, and has low biocompatibility.

Dendrigraft poly-L-lysine (DGL) only consists of lysine monomers, which indicates its complete biodegradability and, thus, the prevention of cumulative cytotoxicity. DLG is favorable for biomedical applications and possesses similar transfection properties to PEI. Therefore, we newly constructed a biodegradable anionic ternary complex consisting of pDNA, DGL, and γ-PGA (Kodama et al., 2014). A previous study reported that the ternary complex with DGL exhibited high gene transfection efficiency in the spleen after its intravenous administration (Kodama et al., 2014). Thus, the application of the ternary complex with DGL as a DNA vaccine is expected, similar to the ternary complex with PEI. However, limited information is available both *in vitro* and *in vivo* on gene expression of the ternary complex with DGL in macrophages. Therefore, we herein examined the gene expression characteristics of this biodegradable ternary complex with DGL in macrophages. We then applied the ternary complex with DGL to pUb-M as a melanoma DNA vaccination. We also assessed the acute and liver toxicities of the pUb-M ternary complex with biodegradable DGL administered at an excess pDNA dose in mice.

## 2. Materials and methods

### 2.1. pDna and chemicals

pDNA encoding luciferase (pCMV-Luc), which expresses luciferase, was constructed as previously described (Kodama, Noda, et al., 2018). pUb-M was obtained from Prof. Reisfeld (Xiang et al., 2000). Cy3-pDNA was prepared using Label IT Cy3 Labeling Kit (Takara Bio Inc., Shiga, Japan) according to the instructions.

A DGL compound (Molecular weight: 172,300 Da, fifth-generation) was obtained from COLCOM S.A.S. (Montpellier, France). γ-PGA was kindly obtained from Yakult Pharmaceutical Industry Co., Ltd. (Tokyo, Japan). Fetal bovine serum (FBS) was obtained from Biological Industries Ltd. (Kibbutz Beit Haemek, Israel). Culture reagents, such as antibiotics (streptomycin and penicillin), Opti-MEM I, and RPMI 1640, were purchased from GIBCO BRL (Grand Island, NY, USA).

### 2.2. Complex preparation

**Figure 1.**
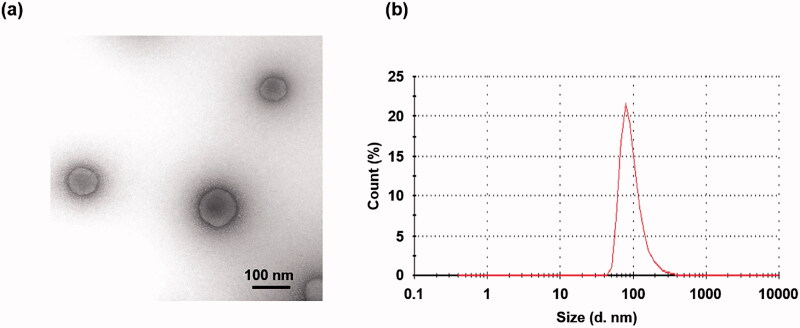
TEM image (a) and dynamic light scattering (DLS) particle size distribution (b) of the ternary complex.

Ternary complexes with DGL were prepared as previously described (Kodama et al., 2014). Briefly, pDNA solution and DGL solution were mixed by thorough pipetting and left for 15 min at room temperature to prepare the binary complexes. To construct the ternary complexes, γ-PGA solution was mixed with the binary complex solution by pipetting and left for another 15 min at room temperature. In this study, we constructed complexes at a theoretical charge ratio, pDNA phosphate:DGL amino:γ-PGA carboxylate = 1:6:8. The configuration of the ternary complex with DGL was observed using transmission electron microscopy as previously reported (Kodama et al., 2017) (Figure 1). The particle size of the ternary complex was observed to be about 100 nm from the TEM images. The particle size and ζ-potential of the ternary complex with DGL measured with a Zetasizer Nano ZS (Malvern Instruments, Ltd., Malvern, United Kingdom) were 93.0 ± 5.8 nm (*n* = 3) and −20.5 ± 0.7 mV (*n* = 3), respectively.

### 2.3. Cells

B16-F10 cells were provided from the Cell Resource Center for Biomedical Research (Tohoku University, Japan). RAW264.7 mouse macrophage cells were purchased from the ATCC (Manassas, VA, USA). B16-F10 cells that express luciferase (B16-F10-Luc cells) were prepared as previously reported (Kurosaki et al., 2013).

### 2.4. In vitro study

RAW264.7 cells were seeded on 24-well plates for 24 h at a density of 1.0 × 10^4^ cells per well in RPMI1640 medium. RPMI1640 medium was then replaced with Opti-MEM I medium and cells were treated with the ternary complex with DGL (including 1 µg pCMV-Luc) and naked pCMV-Luc. Two hours after transfection, Opti-MEM I was changed with RPMI1640 medium. Cells were cultured for 22 h, washed with phosphate-buffered saline, and then treated with lysis buffer. Luciferase activity of lysate was measured as previously described (Kodama et al., 2014).

To visualize the uptake of the complexes, RAW264.7 cells were transfected with Cy3-pDNA or the ternary complex with DGL containing Cy3-pDNA, as described above. After 22 h incubation, the amount of Cy3-pDNA in the cells was characterized using fluorescence microscopy (200× magnification) and quantified by a microplate reader (Infinite-200Pro M-Plex, Tecan Japan Co., Ltd., Kanagawa, Japan).

### 2.5. Cytotoxicity test

RAW264.7 cells were seeded on 96-well plates at a density of 3.0 × 10^3^ cells per well. The cells were treated with naked pDNA, commercial vector, or ternary complex containing 1 µg of pDNA for 2 h. Then, the medium was replaced with 100 µL culture medium and incubated for another 22 h at 37 °C. After incubation, cell viability was determined using a Cell Counting Kit-8 (Dojindo Laboratories, Kumamoto, Japan). The results showed as percentages of the value for the untreated cells (control).

### 2.6. Animals

ddY mice and C57BL/6 mice (5-week-old male) were obtained from Japan SLC (Shizuoka, Japan). All animal experimental procedures were performed in accordance with the Guidelines for Animal Experimentation of Nagasaki University. All animal experiments in the present study were approved by the Institutional Animal Care and Use Committee of Nagasaki University.

### 2.7. *In vivo* study

The ternary complex with DGL (containing 60 µg pCMV-Luc) was administered to the tail vein of mice to investigate gene expression efficiency. Six hours after administration, the tissues (liver, kidneys, spleen, heart, and lungs) of mice were removed and homogenized with lysis buffer. Centrifugation was performed at 21,880 × *g* for 5 min, and the supernatants obtained were used to measure luciferase activities. Luciferase assays were conducted on tissue homogenate samples as previously described (Kodama et al., 2014).

Mice were treated with clodronate liposomes to deplete macrophages in the liver and spleen 24 h prior to the administration of the ternary complex with DGL. Empty liposomes (control liposomes) were used as the control. Six hours after its administration, the liver and spleen of mice were dissected. Luciferase activities in the liver and spleen were measured as described above. In vivo imaging was observed as previously reported (Kodama et al., 2020).

### 2.8. Immunization of mice and assessment of lung metastasis or tumor growth

C57BL/6 mice were intravenously injected with 5% dextrose solution as the control, naked pUb-M, the ternary complex with DGL including pCMV-Luc (pCMV-Luc complex), or the ternary complex with DGL including pUb-M (pUb-M complex) 4 times every 2 weeks at a pDNA dose of 60 µg.

In the assessment of tumor growth, mice were subcutaneously injected with B16-F10 cells (1 × 10^5^ cells) 2 weeks after the last immunization to monitor tumor growth. Tumor volume (*V* mm^3^) was calculated using the following formula:

$$V=\left({\left(\text{Minor axis}\right)}^{2}\times \text{Major axis}\right)/2.$$

In the evaluation of lung metastasis, mice were administered B16-F10-Luc cells (1 × 10^5^ cells) via the tail vein 2 weeks after the last immunization and lung metastasis was monitored. Three weeks after administration, the lungs were dissected, and their luciferase activity was measured as described above.

### 2.9. Toxicological assessments

Mice were intravenously injected with the control or pUb-M complex. Blood samples were collected 6 h later. The serum activities of alanine aminotransferase (ALT) and aspartate aminotransferase (AST) were assessed using commercial biochemical assay kits (FUJIFILM Wako Pure Chemical Corporation, Osaka, Japan).

To examine the acute toxicity of the pUb-M ternary complex, 200 µg (1.5 mL per mouse) of the pUb-M ternary complex or the same volume of 5% dextrose solution was intravenously injected slowly. Twenty-four hours after administration, the number of surviving mice was counted.

### 2.10. Statistical analysis

The significance of differences was assessed using the Student’s *t*-test for two groups. Multiple comparisons were performed using Dunnett’s pairwise multiple comparisons *t*-test.

## 3. Results

### 3.1. Cellular uptake and transgene efficiency of the ternary complex with DGL containing pCMV-Luc in vitro

**Figure 2.**
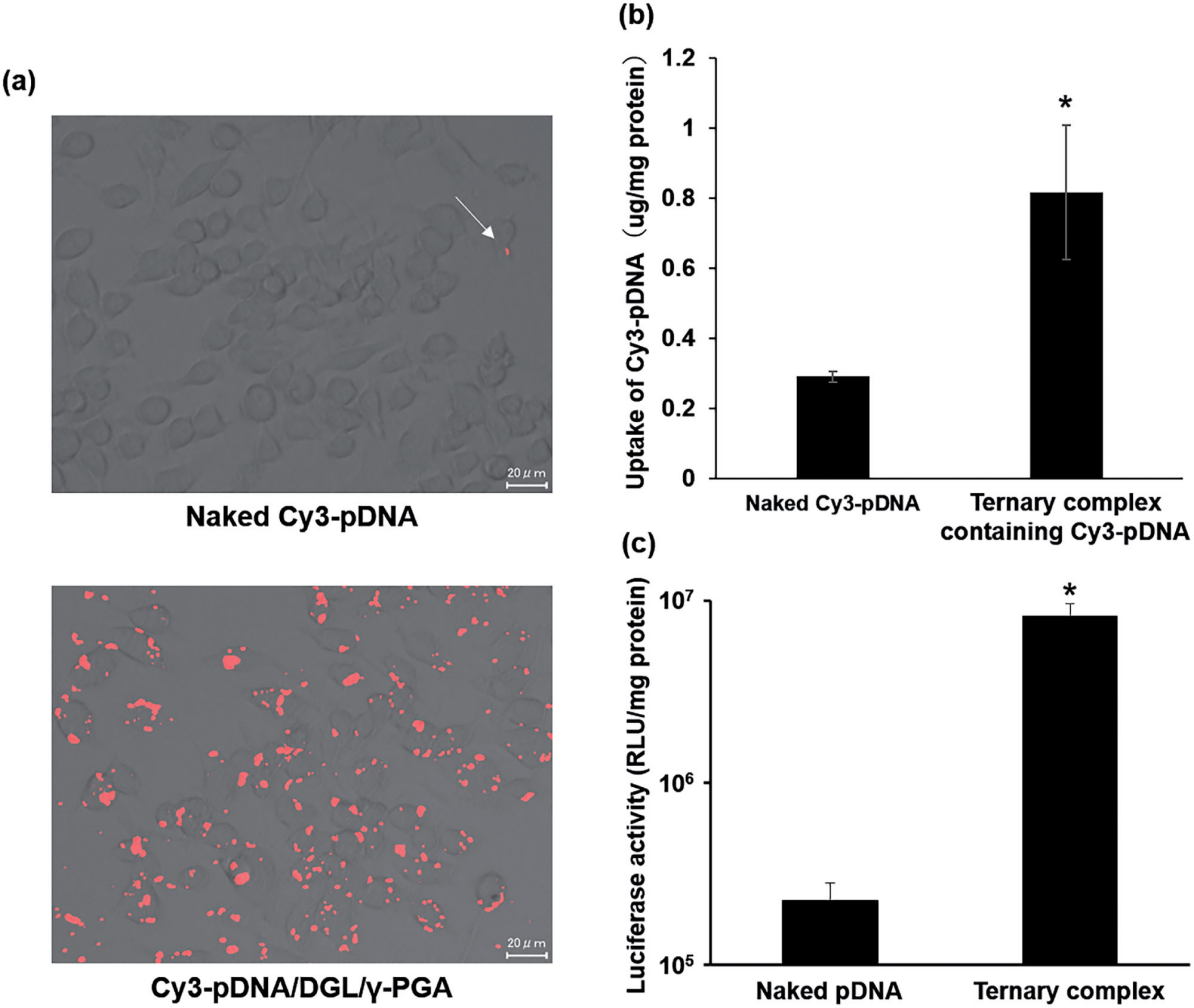
Cellular uptake (a, b) and transgene efficiency (c) of the ternary complex in RAW264.7 cells. RAW264.7 cells were transfected with Cy3-pDNA or the ternary complex with DGL containing Cy3-pDNA. After 22 h incubation, the amount of Cy3-pDNA in the cells was characterized using fluorescence microscopy (a) and quantified by a microplate reader (b). RAW264.7 cells were transfected with naked pCMV-Luc or the ternary complex containing pCMV-Luc. Twenty-two hours after transfection, luciferase activity was evaluated (c). Each bar was the mean ± S.E. *p < .05 vs. control.

RAW264.7 cells were transfected with naked Cy3-pDNA or the ternary complex containing Cy3-pDNA to allow the uptake to be visualized. As a result, naked Cy3-pDNA was not taken up by RAW264.7 cells, although the ternary complex was well taken up by RAW264.7 cells (Figure 2(a,b)).

The ternary complex with DGL containing pCMV-Luc and naked pCMV-Luc were transfected into RAW264.7 cells. The transgene efficacy of the ternary complex with DGL was approximately 70-fold higher than that of naked pCMV-Luc in RAW264.7 cells (Figure 2(c)).

### 3.2. Cytotoxicity of the ternary complex with DGL containing pCMV-Luc in vitro

**Figure 3.**
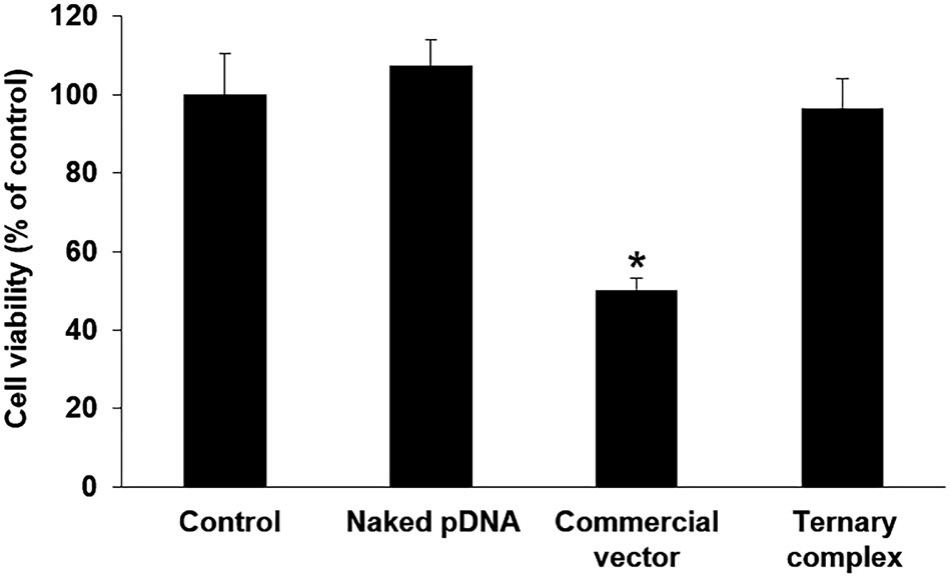
Cytotoxicity of the ternary complex in RAW264.7 cells. RAW264.7 cells were transfected with naked pDNA or the ternary complex containing pDNA. After incubation, cell viability was determined using a Cell Counting Kit-8. Each bar was the mean ± S.E. *p < .05 vs. control.

To examine the cytotoxicity of the ternary complex, RAW264.7 cells were treated with naked pCMV-Luc, commercial vector containing pCMV-Luc, or ternary complex containing pCMV-Luc, and the viability of the cells were evaluated. Commercial vector showed strong cytotoxicity, although ternary complex did not show cytotoxicity as well as naked pDNA (Figure 3).

### 3.3. Transgene efficiency of the ternary complex with DGL containing pCMV-Luc in vivo

**Figure 4.**
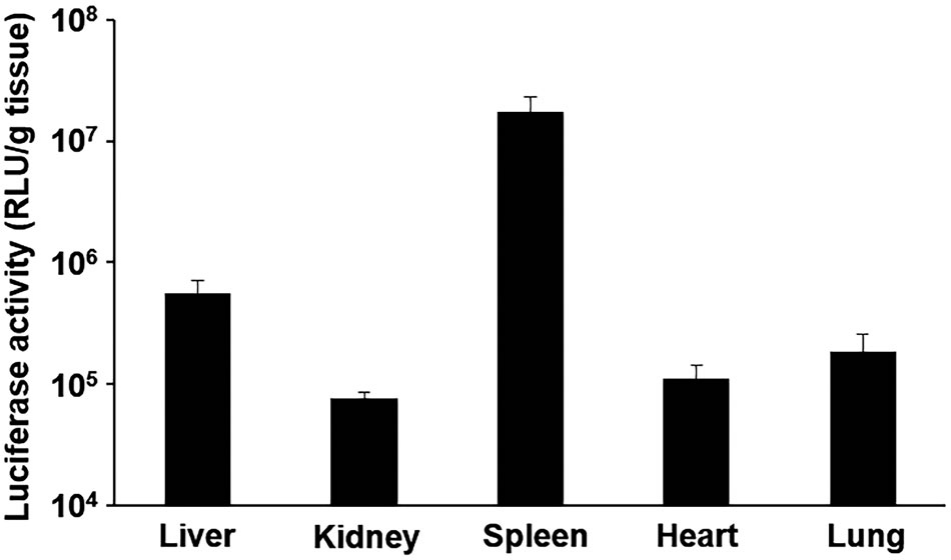
Transgene efficiency of the ternary complex in mice. Mice were intravenously administrated the ternary complex containing pCMV-Luc. Six hours after its administration, luciferase activities in the liver, kidneys, spleen, heart, and lungs were evaluated. Each bar was the mean ± S.E.

The luciferase activities of tissues were assessed 6 h after the tail vein injection of the ternary complex with DGL into mice (Figure 4). The ternary complex with DGL exhibited high luciferase activities of more than 10^7^ RLU/g tissue in the spleen.

**Figure 5.**
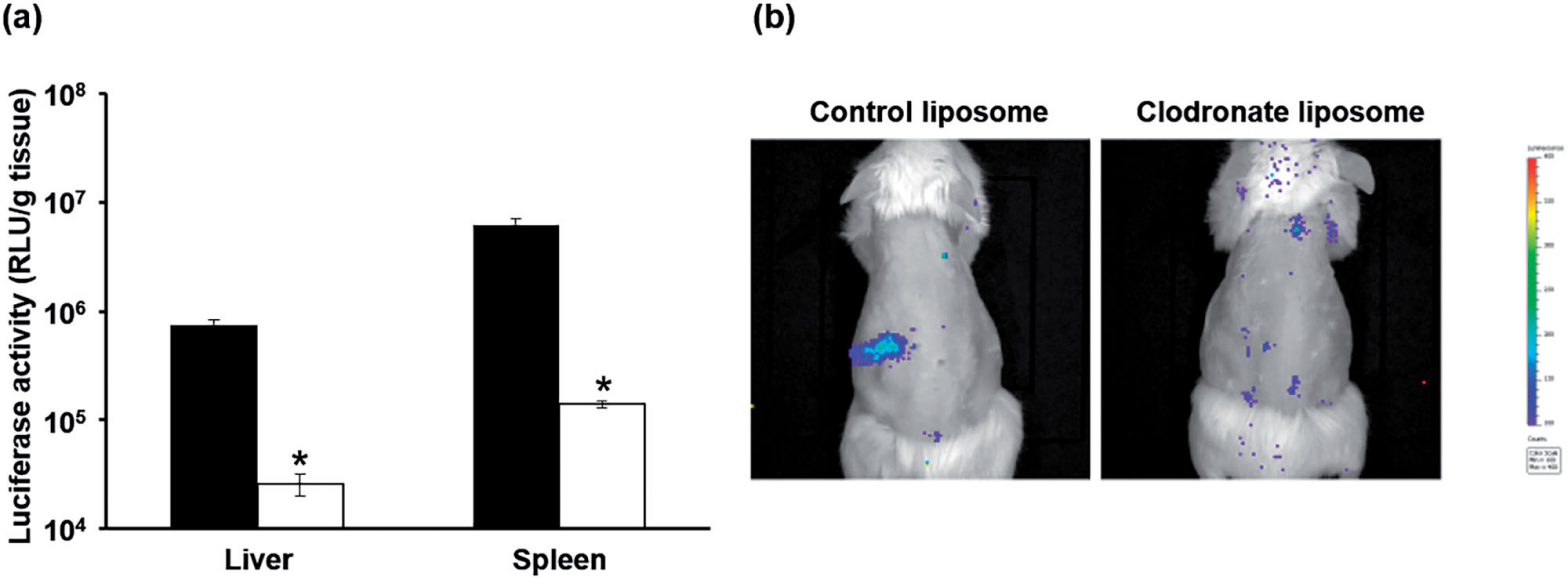
Transgene efficiency of the ternary complex with or without clodronate liposomes in mice. Mice were treated with clodronate liposomes to deplete macrophages in the liver and spleen 24 h prior to the administration of the ternary complex with DGL. Empty liposomes were used as the control. Six hours after its administration, transgene efficiencies were evaluated by luciferase activity in the liver and spleen (a) and IVIS imaging (b). Each bar was the mean ± S.E. *p < .05 vs. control.

Mice were pretreated with clodronate or control liposomes 24 h prior to the tail vein administration of the ternary complex with DGL. The gene expression levels of the liver and spleen were assessed 6 h after its administration. Luciferase activities in the liver and spleen were significantly lower in mice treated with clodronate liposomes than in those treated with control liposomes (Figure 5).

### 3.4. Suppressive effects of the ternary complex with DGL containing pUB-M on melanoma growth

To evaluate the immune response, intradermal transplant and metastatic model mice were treated with 5% dextrose solution, naked pUb-M, pCMV-Luc ternary complex, and pUb-M ternary complex 4 times every 2 weeks.

**Figure 6.**
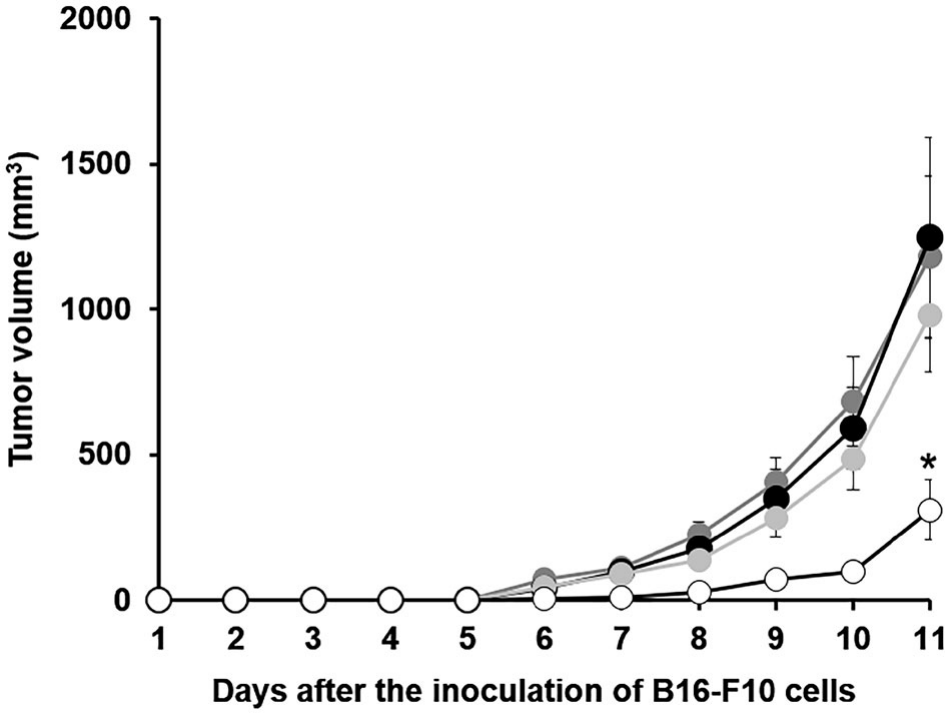
Tumor growth for intradermal transplant model mice intravenously injected with various vaccines. Mice were subcutaneously injected with B16-F10 cells 2 weeks after the last immunization to monitor tumor growth. Tumor volume (*V* mm^3^) was calculated using the following formula: *V* = ((Minor axis)^2^ × Major axis)/2. Each point was the mean ± S.E. *p < .05 vs. control. •: control, •: naked pUb-M, •: pCMV-Luc ternary complex, ○: pUb-M ternary complex

Naked pUb-M and pCMV-Luc ternary complex did not suppress tumor growth in the intradermal transplant model or in the control. In contrast, the pUb-M ternary complex significantly inhibited tumor growth (Figure 6).

**Figure 7.**
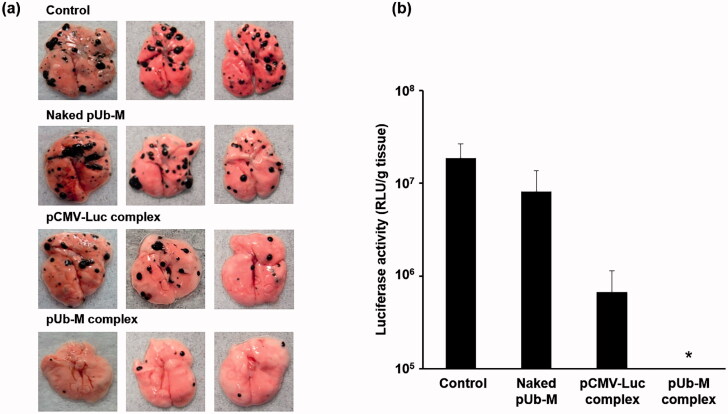
Lung metastasis for metastatic model mice intravenously injected with various vaccines. Two weeks after the last immunization, B16-F10-Luc cells were injected intravenously into mice. Photograph of a B16-F10-derived pulmonary metastatic tumor 3 weeks after the tumor injection in mice immunized by each transfection method (a) Pulmonary metastatic tumors 3 weeks after the tumor injection were evaluated by luciferase activity (b) (*n* = 3, each value represents the mean ± S.E.). Each bar was the mean ± S.E. *p < .05 vs. control.

In the metastatic model, lung metastasis was not inhibited by control and naked pUb-M (Figure 7), whereas it was significantly suppressed by the pUb-M ternary complex. The pCMV-Luc ternary complex also slightly inhibited lung metastasis.

### 3.5. Toxicity of the ternary complex with DGL containing pUb-M *in vivo*

**Figure 8.**
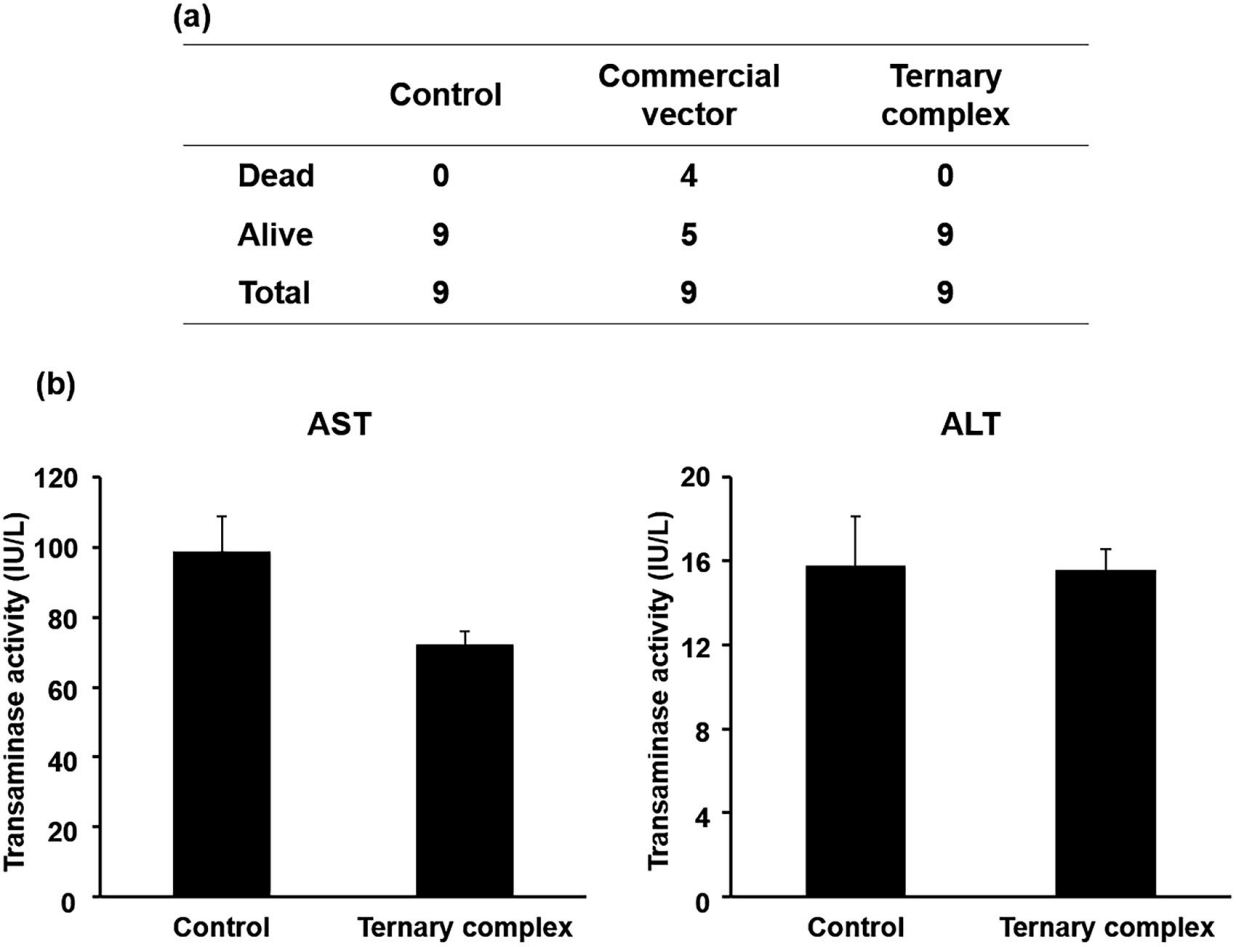
(a) Death of mice 24 h after being treated with the complex. (b) ALT and AST levels in mice 6 h after injected with 5% glucose (control) or the ternary complex.

The *in vivo* acute toxicity of an excess amount of the pUb-M ternary complex with DGL at an excess pDNA dose was evaluated based on the survival rate of mice 24 h after its tail vein injection. All mice survived the injection of the pUb-M ternary complex with DGL, whereas 4 out of 9 mice died after the injection of a commercial vector (Figure 8(a)).

The *in vivo* liver toxicity of the pUb-M ternary complex with DGL was evaluated based on the measurement of liver transaminase levels. Serum AST and ALT levels were normal in mice injected with the pUb-M ternary complex with DGL at an excess pDNA dose (Figure 8(b)).

## 4. Discussion

Melanoma is uniquely and closely related to the immune system, and lymphocyte infiltration often indicates an attempt to eliminate the tumor by the immune system (Agarwala, 2015). Melanoma cells selectively express several differentiation antigens (Hodi, 2006) that may serve as targets for vaccine strategies. Although there are several different vaccine strategies (Lee et al., 2015), plasmid immunization with a gene encoding a tumor antigen is a relatively new strategy for inducing cellular and humoral immune responses (Yuan et al., 2009). Therefore, a DNA vaccine for melanoma is considered to be suitable for preventing metastasis and suppressing tumor growth. A promising strategy to markedly enhance DNA vaccine efficacy is the targeting of a DNA vaccine to APCs (Wang et al., 2011). We previously reported that a ternary complex with DGL containing pUb-M suppressed the lung metastasis of B16-F10 after its pulmonary administration (Kodama et al., 2020). Pulmonary administration is expected to become an appropriate route to extend vaccination coverage because of its more convenient administration and lower risks than routes using needles. On the other hand, systemic administration is a common vaccine administration method that has the advantages of high efficacy and safety. The development of a systemically administrable vaccine with reliable adherence and high efficacy is desired. We previously constructed a ternary complex with DGL, which decreased the toxicity of cationic complexes while maintaining high transgene efficiency (Kodama et al., 2014). The ternary complex was stably included pDNA and induced strong transgene efficiency in the spleen after its tail vein administration (Kodama et al., 2014). Therefore, we herein examined vaccine effects following the intravenous administration of the ternary complex with DGL containing pUb-M.

In order for a DNA vaccine to be effective, it must be taken up by APCs and express the gene encoding pDNA. The ternary complex with DGL showed strong gene expression in the spleen after its tail vein injection (Figure 2). We previously reported that a ternary complex with PEI exhibited high transgene efficiency in the splenic marginal zone (Kurosaki et al., 2013). The marginal zone is a site that is abundant in APCs. We hypothesized that the ternary complex accumulated in macrophages and dendritic cells and exerted vaccine effects. We investigated whether the ternary complex with DGL showed cellular uptake and gene expression in RAW264.7 cells. The results obtained revealed that the ternary complex with DGL was well taken up by cells and shower stronger gene expression than naked pDNA (Figure 2). Also, the ternary complex showed no cytotoxicity in RAW264.7 cells (Figure 3). In addition, we investigated transgene efficiency after the intravenous administration of the ternary complex with DGL following the transient depletion of macrophages by an intravenous injection of clodronate liposomes as previously reported (van Rooijen & van Kesteren-Hendrikx, 2003). The results obtained showed that the transgene efficiency of the ternary complex with DGL in the spleen and liver was significantly decreased by the depletion of macrophages (Figure 4). Therefore, the ternary complex with DGL may have accumulated in splenic macrophages and exhibited gene expression. Previous studies reported the selective uptake of several anionic polymers by macrophages via binding with the scavenger receptor (SR) family (Gough & Gordon, 2000; Martens et al., 2006). Therefore, the ternary complex with DGL may be taken up by macrophages in the spleen via SR; however, further studies are needed to elucidate the underlying mechanisms. Since we developed a ternary complex containing a PyGPI8p-transamidase-related protein-encoding plasmid as a malaria DNA vaccine using the ternary complex with PEI, which achieved complete protection against a lethal challenge in a previous study (Cherif et al., 2014), the ternary complex with DGL may be suitable as a DNA vaccine carrier.

To evaluate the immune induction effects of the ternary complex with DGL as a DNA vaccine carrier, we evaluated the effects of the ternary complex with DGL containing pUb-Μ on B16-F10 cell-derived tumor growth. Transplanted tumors increased in size in 5 days, and some mice died 12 days after tumor transplantation. However, the administration of the pUb-M ternary complex significantly inhibited tumor growth (Figure 4). Systemic immunity may have been activated by the pUb-M ternary complex. However, naked pUb-M and the pCMV-Luc ternary complex did not inhibit tumor growth. These results suggest that the intravenous DNA vaccination of the pUb-M ternary complex is suitable for the inhibition of tumor growth.

A previous study reported that B16-F10 cells metastasized to the lungs after their injection into the lateral tail vein (SiddikuzzamanGrace, 2012). Previous studies established a lung metastasis model with B16-F10 cells intravenously administered to C57BL/6 mice (Gautam et al., 2000; Liu et al., 2013). We used B16-F10-Luc cells as a marker to analyze the tissue deposition of tumor cells, as previously described (Hyoudou et al., 2004, 2006). Mice intravenously administered B16-F10-Luc cells died of lung metastases. However, the vaccination with the pUb-M ternary complex strongly inhibited metastasis to the lungs (Figure 6). In order for the ternary complex with DGL to exert strong cancer vaccine effects, it is important to effectively activate Th1 immunity and induce CTLs with strong antitumor activities (Dredge et al., 2002). We previously reported that a ternary complex with the PEI-containing malaria DNA vaccine significantly increased the levels of IgG and its subtypes as well as the production of Th1 and Th2 type cytokines (Cherif et al., 2014). In addition, the ternary complex with DGL was taken up by macrophages after its pulmonary administration, induced the secretion of Th1 cytokines from splenic cells, and suppressed lung metastasis (Kodama et al., 2020). Therefore, the ternary complex appears to induce the secretion of Th1 cytokines and suppress lung metastasis. In addition, weak therapeutic effects against lung metastasis were observed for the ternary complex with DGL containing pCMV-Luc (Figure 6). The pDNA-containing CpG motif has been shown to induce inflammatory reactions through toll-like receptors (Hemmi et al., 2000), which may weakly suppress lung metastasis. On the other hand, control or naked pUb-M did not suppress lung metastasis. These results indicate that a nano-vaccination that delivers APC cells using a ternary complex with DGL is useful.

Commercial transfection reagents with cationic charges generally exhibit strong cytotoxicity and hematotoxicity (Lv et al., 2006). These toxicities were found to be dose-dependent, which may explain why they have not yet been used in clinical studies (Lv et al., 2006). We previously confirmed that the ternary complex with DGL did not exhibit cytotoxicity or hematotoxicity (Kodama et al., 2014). In the present study, the commercial transfection reagent exhibited strong acute toxicity and approximately 44% of mice administered a large dose subsequently died. The pUb-M ternary complex did not exhibit acute toxicity or cause mice to die (Figure 7). Furthermore, although cationic vectors have been shown to induce hepatic dysfunction after their intravenous administration (Chollet et al., 2002), the pUb-M ternary complex did not cause liver toxicity in the present study. These results suggest that the ternary complex with DGL is safer than the commercial transfection reagent.

In conclusion, a novel biodegradable ternary complex with DGL has potential as a DNA vaccine formulation. The pUb-M ternary complex may suppress the growth and metastasis of B16-F10-derived tumors. The ternary complex with DGL is effective and safe for DNA vaccination and has potential as a platform for vaccines to prevent melanoma. The present results offer valuable basic information for the development of melanoma DNA vaccines. Further study such as the apoptosis and necrosis analysis about the cell death and damage in the tumor tissues will be necessary in the future.
